# Serial Excision of Congenital Melanocytic Nevi

**DOI:** 10.4103/0974-2077.41151

**Published:** 2008-01

**Authors:** Vinod K Jain, Mahendra K Singhi, Rajiv Goyal

**Affiliations:** *Department of Dermatology, STD and Leprosy, SN Medical College, Jodhpur, Rajasthan, India*

**Keywords:** Congenital melanocytic nevus, Serial excision, Recurrence

## Abstract

Congenital melanocytic nevus needs to be excised for aesthetic reasons and concerns regarding its malignant potential. Many methods like surgical resection, dermabrasion, curettage, chemical peeling, laser resurfacing, etc., are available for treatment. We hereby report the efficacy of excision of nevi by serial excision.

## INTRODUCTION

Congenital melanocytic nevus (CMN) is found in approximately 1% of newborn infants.[1] Main factors determining its management are risk of malignancy[2] and the aesthetic consequences. These factors are themselves dependant on the size and localization of the nevi. Main modalities of the treatment are surgical resection (with the help of skin graft or cutaneous expansion or mobilization in giant nevi), dermabrasion and curettage, chemical peeling, laser resurfacing, etc.[1–6] We have found good cosmetic result and absence of recurrence with serial excision in medium size nevi and here by report our findings.

## MATERIALS AND METHODS

Study included five female patients (age 8-24 years) having CMN over forehead (two patients), left cheek (one patient), chin (one patient) and forearm (one patient). The size of the nevi varied from 5 to 8.5 cm diameter (average 6.7 cm). All the lesions were covered with dark-brown to black, coarse terminal hairs. Routine laboratory investigations were done, xylocaine sensitivity was performed, close-up photographs were taken and consent was obtained. Preoperative anxiolytic and broad-spectrum antibiotics were started in the previous evening.

After cleansing the area thoroughly with povidone iodine and spirit, the proposed incision line was marked by 1% gentian violet paint. Under local anaesthesia (2% lignocaine with adrenaline 1:200,000), incision was made using scalpel blade, deep up to subcutaneous tissue (little deeper than the hair follicles). Thorough undermining was done using dissecting scissors. After achieving complete haemostasis, subcutaneous interrupted sutures using synthetic absorbable suture (Vicryl™ 4-0 or 5-0 with cutting needle) was applied. Skin was stitched with non-absorbable polypropylene monofilament suture (Prolene™ 4-0 to 6-0 suture with cutting needle). Wound was dressed by composite dressing using mupirocin 1% ointment. Care was taken to relax the stitch line as much as possible by applying adhesive tape. Dressing as well as sutures were removed after five to seven days. Sterile medicated adhesive dressing (*viz*, Band-aid^®^, Steri-strips^®^) was applied and was kept at least for 3-4 weeks. A second surgical excision was done after 2 months.

## RESULTS

Surgery in all cases was uneventful except for breakthrough of two sutures in one case on first sitting because of excessive tension in sutures, a small haematoma in one case which was removed after removing two stitches. Fifty to seventy percentage of total nevi was removed in first sitting and rest was removed on second sitting. Patients and attendants felt reassured after the result of first sitting, quite satisfied after second sitting and were very happy after 2-3 months when stitch marks also faded away on its natural course. No topographical deformities like mis-shapen chin, asymmetry of the part, etc., were noticed.

## DISCUSSION

Removal of a medium size CMN over exposed parts, especially over face is warranted for its cosmetic, embarrassment rather than for its potential to cause malignancy. Out of the many methods available (*viz*, surgical resection, with or without skin grafting, cutaneous expansion or mobilization) cosmetic result of surgical resection with primary suturing is always preferable whether done in one stage or 2-3 stages. i.e., serial excision.[2] This is technically less demanding and can be performed even by a novice cutaneous surgeon if basic principles of cosmetic surgery are taken care of. We have found this technique to be superior to treatment of CMN by CO_2_ laser. Our earlier two cases who were treated by CO_2_ laser presented with recurrence within 6 months, while the five patients in whom serial excision was done revealed no signs of recurrence even after 1-1.5 years follow-up. Cause of recurrence in patients treated with CO_2_ laser was probably the residual nevus cells in the lower dermis. Hence, we must excise the nevus in its full depth so that it comes out enmasse.

## References

[1] Michel JL, Chalencon F, Gentil-Perret A, Fond L, Montélimard N, Chalencon V (1999). Congenital pigmented nevus: Prognosis and therapeutic possibilities. Arch Pediatr.

[2] Kruk JJ, Lewandowicz E, Rykala (1999). Surgical treatment of pigmented melanocytic nevi depending upon their size and location. J Acta Chir Plast.

[3] Kay AR, Kenealy J, Mercer NS (1998). Successful treatment of a giant melanocytic nevus with the high energy pulsed CO_2_ laser. Br J Plast Surg.

[4] Hopkins JD, Smith AW, Jackson IT (2000). Adjunctive treatment of congenital pigmented nevi with phenol chemical peel. Plast Reconstr Surg.

[5] Gambichler T, Senger E, Rapp S, Alamouti D, Altmeyer P, Hoffmann K (2000). Deep shave excision of macular melanocytic nevi with the razor blade biopsy technique. Dermatol Surg.

[6] Hamilton SA, Kirk J, Morris AM (2001). Long-term results of surgical excision and skin grafting for a giant hairy nevus of the face: Time for a return to conventional wisdom. Br J Plast Surg.

